# Hematopoietic stem cell transplantation in children and adolescents with GATA2-related myelodysplastic syndrome

**DOI:** 10.1038/s41409-021-01374-y

**Published:** 2021-07-09

**Authors:** Rachel Bortnick, Marcin Wlodarski, Valerie de Haas, Barbara De Moerloose, Michael Dworzak, Henrik Hasle, Riccardo Masetti, Jan Starý, Dominik Turkiewicz, Marek Ussowicz, Emilia Kozyra, Michael Albert, Peter Bader, Victoria Bordon, Gunnar Cario, Rita Beier, Johannes Schulte, Dorine Bresters, Ingo Müller, Herbert Pichler, Petr Sedlacek, Martin G. Sauer, Marco Zecca, Gudrun Göhring, Ayami Yoshimi, Peter Noellke, Miriam Erlacher, Franco Locatelli, Charlotte M. Niemeyer, Brigitte Strahm, for EWOG-MDS

**Affiliations:** 1 Department of Pediatrics and Adolescent Medicine, Division of Pediatric Hematology and Oncology, Medical Center, Faculty of Medicine, University of Freiburg, Freiburg, Germany; 2 Department of Hematology, St. Jude Children’s Research Hospital, Memphis, TN USA; 3 Princess Maxima Center, Diagnostic Laboratory/DCOG Laboratory, Utrecht, The Netherlands; 4 Department of Pediatric Hematology-Oncology and Stem Cell Transplantation, Ghent University Hospital, Ghent, Belgium; 5 Department of Pediatrics, St. Anna Children’s Hospital, Medical University of Vienna, Vienna, Austria; 6 Department of Pediatrics, Aarhus University Hospital Skejby, Aarhus, Denmark; 7 Department of Pediatric Oncology and Hematology, University of Bologna, Bologna, Italy; 8 Department of Pediatric Hematology and Oncology, Charles University and University Hospital Motol, Prague, Czech Republic; 9 Department of Pediatric Oncology/Hematology, Skåne University Hospital, Lund, Sweden; 10 Department of Bone Marrow Transplantation, Oncology and Hematology, Wroclaw Medical University, Wroclaw, Poland; 11 Department of Pediatrics, Dr. von Hauner Children’s Hospital, University Hospital, LMU, Munich, Germany; 12 Department for Children and Adolescents, Division for Stem Cell Transplantation and Immunology, University Hospital Frankfurt, Frankfurt am Main, Frankfurt, Germany; 13 Department of Pediatrics, Christian-Albrechts-University Kiel and University Medical Center Schleswig-Holstein, Kiel, Germany; 14 Department of Pediatrics and Adolescent Medicine, Division of Pediatric Hematology and Oncology, University Hospital of Essen, Essen, Germany; 15 Department of Pediatric Oncology, Hematology and Stem Cell Transplantation, Charité University Medicine Berlin, Berlin, Germany; 16 Princess Máxima Center for Pediatric Oncology, Utrecht, The Netherlands; 17 Department of Pediatric Hematology and Oncology, University Medical Center Hamburg-Eppendorf, Hamburg, Germany; 18 Pediatric Hematology and Oncology, Hannover Medical School, Hannover, Germany; 19 Pediatric Hematology/Oncology, Fondazione IRCCS Policlinico San Matteo, Pavia, Italy; 20 Department of Human Genetics, Hannover Medical School, Hannover, Germany; 21 German Cancer Consortium (DKTK), Heidelberg and Freiburg, Freiburg, Germany; 22 Department of Pediatric Hematology and Oncology, IRCCS Ospedale Pediatrico Bambino Gesù, Sapienza, University of Rome, Rome, Italy; 23 Present Address: Department of Pediatrics, University Hospital Salzburg, Paracelsus Medical University, Salzburg, Austria

**Keywords:** Stem-cell therapies, Paediatrics

## Abstract

GATA2 deficiency is a heterogeneous multi-system disorder characterized by a high risk of developing myelodysplastic syndrome (MDS) and myeloid leukemia. We analyzed the outcome of 65 patients reported to the registry of the European Working Group (EWOG) of MDS in childhood carrying a germline *GATA2* mutation (*GATA2*^mut^) who had undergone hematopoietic stem cell transplantation (HSCT). At 5 years the probability of overall survival and disease-free survival (DFS) was 75% and 70%, respectively. Non-relapse mortality and relapse equally contributed to treatment failure. There was no evidence of increased incidence of graft-versus-host-disease or excessive rates of infections or organ toxicities. Advanced disease and monosomy 7 (−7) were associated with worse outcome. Patients with refractory cytopenia of childhood (RCC) and normal karyotype showed an excellent outcome (DFS 90%) compared to RCC and −7 (DFS 67%). Comparing outcome of *GATA2*^mut^ with *GATA2*^*wt*^ patients, there was no difference in DFS in patients with RCC and normal karyotype. The same was true for patients with −7 across morphological subtypes. We demonstrate that HSCT outcome is independent of *GATA2* germline mutations in pediatric MDS suggesting the application of standard MDS algorithms and protocols. Our data support considering HSCT early in the course of GATA2 deficiency in young individuals.

## Introduction

Myelodysplastic syndrome (MDS) in young individuals consists of a heterogeneous group of hematopoietic disorders characterized by ineffective hematopoiesis, peripheral blood cytopenia, cellular dysplasia and a high risk of progression to acute myeloid leukemia (AML). In contrast to older adults, in whom MDS is associated with age-related somatic mutations, MDS in young patients is often associated with genetic syndromes predisposing to myeloid neoplasia. Next to the well-known inherited bone marrow failure syndromes like Fanconi anemia, Shwachman–Diamond syndrome, severe congenital neutropenia, or dyskeratosis congenita, a slew of predisposition syndromes involving genes like *GATA2, SAMD9/SAMD9L, RUNX1, ANKRD26, ETV6, SRP72, ERCC6L2*, and others have recently been uncovered [1–4].

Among these new genetic entities, GATA2 deficiency resulting from heterozygous germline mutations in the gene encoding the zinc-finger transcription factor *GATA2* appears to be the most common predisposing condition for MDS in childhood [5, 6]. Although some patients with germline mutations in *GATA2* (*GATA2*^mut^) have a positive family history, de novo germline mutations have been reported in a majority of children with *GATA2*^mut^ MDS [6]. Despite the observation that the loss of B-cells is a common feature of GATA2 deficiency [7], children with *GATA2* germline mutations often present as MDS without prior infections. In contrast, young adults often display a history of opportunistic infections, slowly progressing bone marrow failure, and subsequent transformation to AML.

The prevalence of myeloid neoplasia in GATA2 deficiency has been estimated to be 75%, with a median age at diagnosis of 19.7 years [8]. Studying a series of 79 *GATA2*^mut^ patients, Donadieu described that more than 80% of patients had developed a hematological malignancy by the age of 40 years; progression from MDS to AML was observed in 16% [5]. Examining a cohort of over 600 individuals with MDS enrolled in the registries of the European Working Group of MDS in Childhood (EWOG-MDS), our group reported a prevalence of *GATA2*^mut^ in 7% of all primary MDS and 15% in advanced primary MDS. *GATA2* germline disease was associated with more advanced MDS type and often accompanied by monosomy 7 [6].

Allogeneic HSCT is the only curative therapy for hematological complications of GATA2 deficiency, and has been shown to eradicate clonal malignancy, restore normal hematopoiesis, clear underlying infections and improve pulmonary function. As GATA2 deficiency is a newly defined disease, HSCT strategies, as well as outcome, have yet to be fully elucidated. In particular, it is unclear whether applying guidelines for HSCT in pediatric MDS results in similar outcome. Most published reports refer to single-patient case studies, small series of primarily adult patients, or patients with immunodeficiency in the absence of clonal disease [9–15]. We have previously observed that 34 individuals with MDS, monosomy 7 and *GATA2*^mut^ had a similar outcome compared to their counterparts with wildtype *GATA2* (*GATA2*^wt^) [6]. Here we expand the analysis to an enlarged cohort with longer follow-up and provide a detailed review of HSCT in young individuals with GATA2 deficiency.

## Methods

### Study population

We identified 66 patients with MDS and *GATA2* germline mutation prospectively enrolled for MDS in the EWOG-MDS registries (EWOG-MDS 98 #NCT00047268, EWOG-MDS 2006 #NCT00662090) who had undergone HSCT at an age of <20 years between 01/1997 and 11/2018. One patient was excluded from the analysis due to missing data. Genetic and/or clinical data from 50 patients had partially been included in previous publications [6, 16]. HSCT procedures had been performed in accordance with EWOG-MDS recommendations (www.ewog-mds-saa.org). MDS was classified based on the 2016 WHO classification for pediatric MDS, and included refractory cytopenia of childhood (RCC), MDS with excess blasts (MDS-EB), MDS-EB in transformation (MDS-EBt), and MDS-related acute myeloid leukemia (MDR-AML) [17]. One patient with myelofibrotic MDS and increased BM blasts was classified as MDS-EBt. Cytogenetic analysis was performed according to standard procedures and described according to the International System for Human Cytogenetic Nomenclature. Karyotypes with sole monosomy 7, and monosomy 7 with one or two additional random aberrations were classified as monosomy 7 and analyzed in one group [18].

Molecular studies to identify *GATA2* mutations were conducted as previously described [6, 16]. In patients enrolled before 2013 *GATA2* testing was performed retrospectively, thereafter the diagnosis of MDS prompted *GATA2* testing independent of the clinical presentation. For the analyses comparing *GATA2*^mut^ to *GATA2*^wt^ patients, we identified 404 *GATA2*^wt^ MDS patients without known underlying predisposition (including *SAMD9/L*) who otherwise fulfilled the study criteria (Supplementary Fig. 1).

All studies were approved by the institutional ethics committees of the respective institutions. Written informed consent was obtained from patients or patients’ guardians. The study was conducted in accordance with the Declaration of Helsinki.

### Statistical analysis

Overall survival (OS) was defined as the time from HSCT to death or last follow-up, disease-free survival (DFS) was defined as the time from HSCT to death, disease recurrence, or last follow-up. The Kaplan–Meier method was used to estimate survival rates, and the two-sided log-rank test was used to evaluate the equality of the survivorship functions in different subgroups. Time-to-event outcome for relapse and non-relapse mortality (NRM) were estimated using cumulative incidence curves, using relapse and NRM as the respective competing risks [19, 20]. Differences in the cumulative incidence functions among groups were compared using Gray’s test [21].

For the analyses comparing *GATA2*^mut^ with *GATA2*^wt^ patients the *χ*^2^ test was used to examine the statistical significance of the association between *GATA2* status and categorized factors. Fisher’s exact test was calculated for 2 × 2 contingency analyses. Nonparametric statistics were used to test for differences in continuous variables in terms of mutational status (Mann–Whitney *U* test).

For multivariate analysis, a cause-specific Cox model was fitted, including all variables with *P* less than 0.1 in the univariate analysis for DFS [22]. The model included the *GATA2* status, karyotype, and highest WHO-type. All *P* values were two-sided, and values < 0.05 were considered to be statistically significant. Software packages SPSS for Windows 25.0.0 (IBM Corp, New York, NY) and NCSS 2004 (NCSS, Kaysville, UT) were used.

## Results

### Characteristics of the cohort

The 65 children and adolescents with GATA2 deficiency had been diagnosed with RCC (*n* = 36), MDS-EB (*n* = 22), MDS-EBt (*n* = 6) or MDR-AML (1) at a median age of 12.8 (4.4–18.6) years. Karyotypes included monosomy 7 (*n* = 44), der (1;7) (*n* = 4), trisomy 8 (*n* = 4), random aberration (*n* = 1) or a normal karyotype (*n* = 12). Forty patients (71%) had additional non-hematological features of GATA2 deficiency (Table 1). Prior to HSCT, 16 patients had progressed to a more advanced stage of MDS and five had received AML-type chemotherapy, resulting in a BM blast count of <5% at the time of HSCT.

**Table 1:** Patient characteristics and transplantation procedure.

Item	Specification	At diagnosis/prior to HSCT		
			*N*	%
Patients			65	100
Gender	Male		34	52
	Female		31	48
Age at diagnosis of MDS	Years, median(range)	12.8 (4.4–18.6)		
GATA2 Type of mutation	Truncating		43	66
	Missense		14	22
	Non-Coding Intron 4		4	6
	Synonymous		3	5
	Whole gene deletion		1	2
MDS subtype at diagnosis	RCC		36	55
	MDS-EB		22	34
	MDS-EBt/ MDR-AML		6 /1	11
Karyotype	Monosomy 7		44	68
	Der (1;7)		4	6
	Trisomy 8		4	6
	Normal^a^		12	19
	Other		1	1
Non-Hematological features	Any		40	71
	Immunedeficiency^b^		24	
	Lymphedema/ hydrocele		13	
	Deafness/hearing impairment		8	
	Urogenital malformations		10	
	Neurocognitive/ behavioral problems		10	
Highest MDS subtype				
(prior to HSCT)	RCC		27	42
	MDS-EB		23	35
	MDS-EBt/ MDR-AML		10/5	23
	**At HSCT**			
Age at HSCT	Years, median (range)	13.5 (4.6-19.9)		
Interval MDS to HSCT	Months, median (range)	5.6 (1.4 – 67)		
Therapy prior to 1st HSCT	No therapy		55	85
	AML-type		5	8
	other		5	8
BM blasts at HSCT	< 5%		34	56
	5–19%		19	31
	≥ 20%		8	13
	*No data*		4	
	**HSCT procedure**			
Donor	MSD		17	26
	MUD (10/10)/(9/10)		24/6	46
	UD (6/6)/(5/6)/(8/10)^c^/ incomplete typing		1/2/6/1	15
	MMFD		8	12
Stem cell source	BM		37	57
	PBSC		19	29
	PBSC (T-cell depleted)		8	12
	CB		1	2
Conditioning regimen	Busulfan- based		35	54
	Treosulfan-based		21	32
	TBI-based		5	8
	Other		4	6
GvHD prophylaxis	MSD (17)	CSA	7	
		CSA/MTX	7	
		ATG/CSA/MTX	3	
	(M)UD (40)	ATG^d^/CSA/MTX^e^	36	
		ATG/CSA	2	
		ATG/tacrolimus	1	
		CSA/MTX	1	
	MMFD (8)	ATG	6	
		ATG/MMF	1	
		Muromonab (OKT3)	1	

*HSCT* Hematopoietic stem cell transplantation, *MDS* Myelodysplastic syndrome, *RCC* Refractory cytopenia of childhood, *MDS-EB* MDS with excess blasts, *MDS-EBt* MDS with excess blasts in transformation, *MDR-AML* MDS-related acute myeloid leukemia, *MSD* matched sibling donor, *MUD* matched unrelated donor, *UD* unrelated donor, *MMFD* mismatched family donor, *BM* bone marrow, *PBSC* peripheral blood stem cells, *CB* cord blood; *TBI* total body irradiation, *ATG/ALG* anti-thymocyte/lymphocyte globuline, *CSA* cyclosporine, *MTX* methotrexate, *MMF* Mycophenolate mofetil.

^a^Including two patients without sufficient metaphases and exclusion of monosomy 7 and trisomy 8 by fluorescence in situ hybridization (FISH).

^b^Defined as frequent infections and/or laboratory evidence of immune deficiency.

^c^Including one patient with an 8/10 HLA matched sibling donor.

^d^Including one patient with alemtuzumab instead of ATG as serotherapy.

^e^Including two patients with MMF instead of MTX.

Patients had undergone HSCT from a matched sibling donor (MSD; *n* = 17), unrelated donor (UD; *n* = 40) or mismatched family donor (MMFD; *n* = 8) at a median age of 13.5 (4.6–19.9) years (Table 1). Stem cell source was BM (*n* = 37), peripheral blood (*n* = 27) or cord blood (*n* = 1). Patients were prepared with a busulfan-based (*n* = 35), treosulfan-based (*n* = 21), total body irradiation-based (*n* = 5) or an alternative conditioning regimen (*n* = 4). Graft-versus-host-disease (GvHD) prophylaxis included cyclosporine ± methotrexate for the majority of MSD-HSCT and additional anti-thymocyte globulin in UD-HSCT.

### Engraftment and GvHD

All patients achieved initial engraftment. Secondary graft failure occurred in four patients (Supplementary Table 1) following MMFD-HSCT (*n* = 2) or MUD-HSCT (*n* = 2) resulting in death in two patients.

**Fig. 1:**
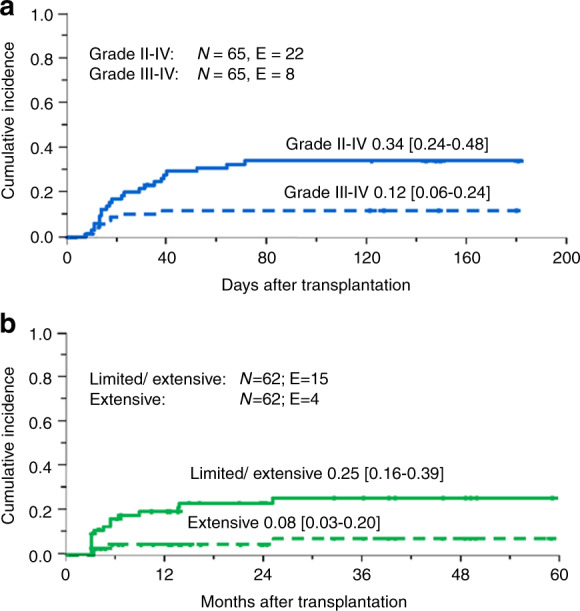
Incidence of acute and chronic GvHD. **A** Cumulative incidence of day 100 grade II–IV and III–IV acute GvHD. **B** Cumulative incidence of chronic GvHD in the 62 patients at risk. N numbers, E events.

The cumulative incidence of acute GvHD (aGvHD) at day 100 was 0.34 [95% CI 0.24–0.48] for grade II–IV and 0.12 [0.06–0.24] for grade III–IV (Fig. 1A). Following MSD-HSCT two patients developed grade III–IV aGvHD (12%), while six patients grafted from an UD experienced grade III–IV aGvHD (15%). None of the patients transplanted from a MMFD had grade II–IV aGvHD (Supplementary Table 1).

Fifteen of the 62 patients at risk (24%) developed chronic GvHD (cGvHD), which scored limited in 11 cases and extensive in the remaining four. The cumulative incidence of overall and extensive cGvHD was 0.25 [0.16-0.39] and 0.08 [0.03-0.20], respectively (Fig. 1B). Among the 16 patients at risk grafted from a MSD, four (25%) developed cGvHD, while nine of 39 patients at risk (23%) developed cGvHD following UD-HSCT. Of the patients transplanted with a MMFD, two out of seven patients at risk developed limited cGvHD (Suppl. Table 1).

### Infections and toxicity

Evaluating the frequency of infections post-HSCT, 49 patients were noted to develop any infection. Forty-one had viral infections, 16 bacterial infections, and 9 patients fungal disease (7 aspergillosis, one candidiasis, one unknown). The most common viral infections were CMV and EBV in 16 and 10 patients, respectively; one patient each developed CMV disease and post-transplant lymphoproliferative disease (Table 2). Adenovirus infection was recorded in four patients.

**Table 2:** Infectious disease post-HSCT.

Type of infections	Number of patients (*N*)
None	16
Bacterial	16
Fungal	9
Viral	41

Type of viral infections	Number of patients (N)
CMV reactivation	16
CMV disease	1
EBV reactivation	10
PTLD	1
Adenovirus	4
Other virus infection	22

*EBV* Epstein–Barr virus, *CMV* cytomegalovirus, *PTLD* post-transplant lymphoproliferative disease.

With respect to non-infectious complications, the rate of complications resulting in toxicity of grade 3 or more according to Common Terminology Criteria for Adverse Events was 43%. Thirteen patients had ≥1 non-infectious complication. Hepatobiliary (16, including 3 veno-occlusive disease) and pulmonary (13) toxicity was most common (Table 3). Four patients experienced neurologic complications, three of which were described as posterior reversible encephalopathy syndrome.

**Table 3:** Non-infectious complications post-HSCT.

Type of complications	Number of patients (*N*)	
Pulmonary toxicity	13	
Liver complications	13	
VOD	3	
Renal complications	6	
Neurological complications	4	
Gastrointestinal complications	3	
Cardiac complications	2	
Transplant-related microangiopathy	3	
Autoimmune hemolytic anemia	1	
Acute pancreatitis	1	

Number of complications	Number of patients (*N*)	%
None	37	57
1 Complication	15	23
2 Complications	9	14
3 Complications	2	3
4 Complications	0	
5 Complications	2	3

*VOD* veno-occlusive disease, *HSCT* hematopoietic stem cell transplantation.

### Overall outcome

**Fig. 2:**
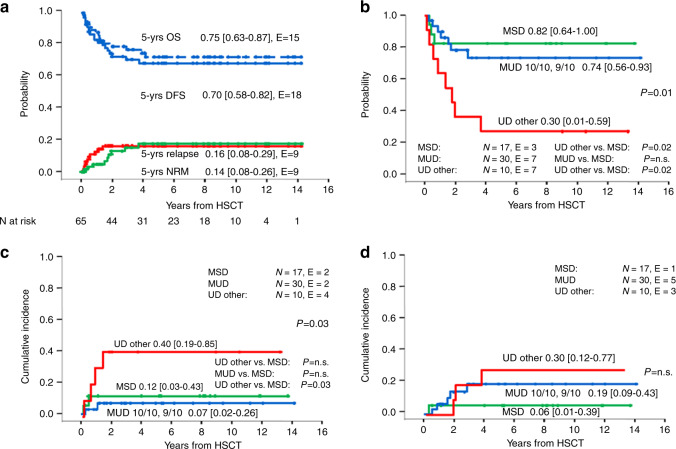
Overall outcome and outcome according to type of donor. **A** Overall and disease-free survival and cumulative incidence of relapse and non-relapse mortality for 65 patients with MDS and *GATA2* germline mutation undergoing HSCT. **B** Disease-free survival, **C** non-relapse mortality and **D** relapse according to type of donor. In the group of eight patients grafted from a mismatched family donor (MMFD) only one non-relapse mortality was observed (data not shown). MSD matched sibling donor, MUD matched unrelated donor (9/10 or 10/10), UD other other unrelated donor, N numbers in subgroup, E events.

Fifty patients were alive 5 years after HSCT, resulting in a Kaplan–Meier estimate of 5-year OS of 0.75 [0.63–0.87] (Fig. 2A). The probability of DFS was 0.70 [0.58–0.82] (Fig. 2A). The cumulative incidence of relapse (CIR) was 0.16 [0.08–0.29] and of NRM 0.14 [0.08–0.26]; Fig. 2A. Nine patients died of transplant-related causes. DFS was comparable for patients transplanted from MUD (0.74 [0.56–0.93]) versus MSD (0.82 [0.64–1.00]), whereas patients transplanted from mismatched UD (UD other) had a significantly lower DFS (0.30 [0.01–0.59]; *p* = 0.01) (Fig. 2B). The latter was primarily due to a significantly higher NRM for UD other of 0.40 [0.19–0.85] compared to 0.12 [0.03–0.43] for MSD and 0.07 [0.02–0.26] for MUD, *p* = 0.03; (Fig. 2C) whereas there was no significant difference in the CIR according to type of donor (Fig. 2D). Of note, of the eight patients transplanted from a MMFD, only one died after secondary graft failure, while the other seven patients are alive and disease-free. In univariate analysis, none of the other transplantation procedure-related variables such as year of HSCT, conditioning regimen, time from diagnosis to HSCT, stem cell source or donor and recipient sex had a significant impact on DFS, NRM, and CIR (Supplementary Table 2).

### Outcome according to MDS type and karyotype

**Fig. 3:**
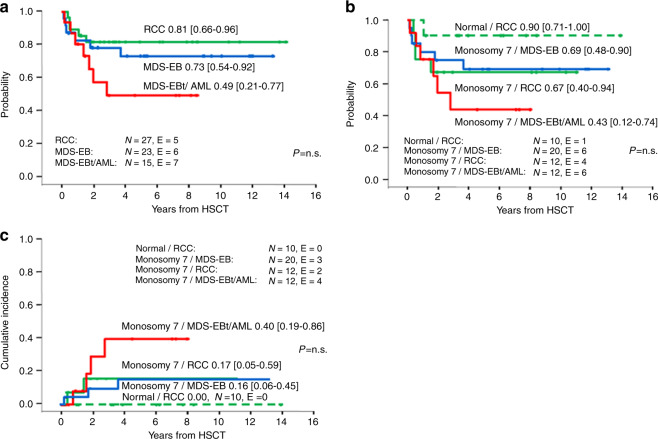
Outcome from HSCT according to MDS subtype and karyotype. **A** Disease-free survival according to most advanced MDS type prior to transplantation, **B** Disease-free survival, and **C** cumulative incidence of relapse according to most advanced MDS type stratified by karyotype. RCC refractory cytopenia of childhood, MDS-EB MDS with excess blasts, MDS-EBt MDS with excess blasts in transformation, AML MDS-related acute myeloid leukemia, N numbers in subgroup, E events.

Patients with a BM blast percentage of >20% at any time prior to HSCT showed a trend toward inferior DFS (0.49 [0.21–0.77]) compared to patients with 5–19% BM blasts (0.73 [0.54–0.92]) or with <5% blasts (0.81 [0.66–0.96]) (*p* = 0.15; Fig. 3A). Similarly, there was a trend toward a higher CIR and NRM (data not shown).

We next assessed the association between karyotype and morphologic diagnosis. Normal karyotype was associated with RCC (10/12 patients with normal karyotype had RCC) and monosomy 7 was associated with advanced MDS (32/38 advanced MDS patients had monosomy 7) (Supplementary Table 3). Thus, we performed a stratified analysis combining MDS type and karyotype. Patients with RCC and normal karyotype showed a superior DFS (0.90 [0.71–1.00]) compared to patients with monosomy seven independent of disease status (RCC 0.67 [0.40–0.94], MDS-EB 0.69 [0.48–0.90], MDS-EBt/MDR-AML 0.43 [0.12–0.74]) (Fig. 3B). While none of the patients with RCC and normal karyotype relapsed, patients with MDS-EBt/MDR-AML and monosomy 7 karyotype showed the highest relapse rate (0.40 [0.19–0.86]) (Fig. 3C).

### Comparison of outcome to MDS without known underlying predisposition syndrome

**Fig. 4:**
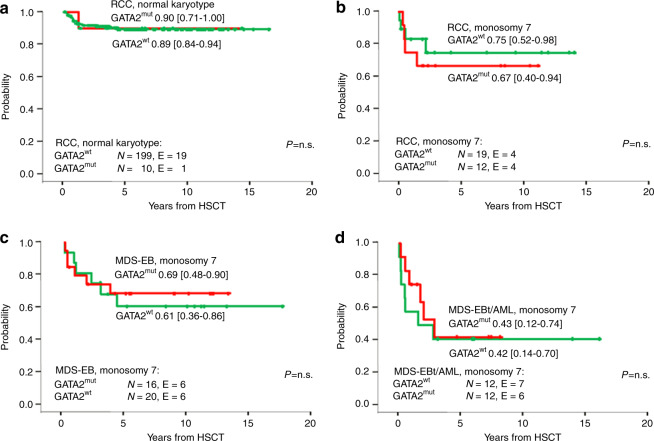
Outcome from HSCT comparing *GATA2*^mut^ and *GATA2*^wt^ patients. Disease-free survival in *GATA2*^mut^ vs *GATA2*^wt^ cohorts for patients **A** with RCC and normal karyotype, **B** RCC and monosomy 7, **C** MDS-EB and monosomy 7 and **D** MDS-EBt/AML and monosomy 7. RCC refractory cytopenia of childhood, MDS-EB MDS with excess blasts, MDS-EBt MDS with excess blasts in transformation, AML MDS-related acute myeloid leukemia, N numbers in subgroup, E events.

Next, we performed an analysis comparing the HSCT outcome of 65 *GATA2*^mut^ patients with a cohort of 404 *GATA2*^wt^ patients registered in EWOG-MDS and transplanted during the same time period (Supplementary Table 3). As expected, *GATA2*^mut^ patients were slightly older, had more advanced disease, and carried a monosomy 7 karyotype more frequently (Supplementary Table 3). At 5 years there was no significant difference in OS (*GATA2*^wt^ 0.82 [0.78–0.86] vs *GATA2*^mut^ 0.75 [0.63–0.87]) and DFS (*GATA2*^wt^ 0.78 [0.74–0.82] vs *GATA2*^mut^ 0.70 [0.58–0.82]). Comparing the outcome of RCC patients with normal karyotype with respect to the presence or absence of a germline *GATA2* mutation, both groups showed nearly identical probabilities of DFS of 90% and 89%, respectively (Fig. 4A). Similarly, there was no significant difference in DFS among patients of any MDS type with monosomy 7 with respect to the presence or absence of GATA2 deficiency (Fig. 4B–D).

In multivariate analysis of variables predicting DFS (including age, karyotype, highest MDS subtype and *GATA2* status), the most important factors were karyotype (monosomy 7 vs. normal; *p* < 0.01) and most advanced MDS type (RCC vs MDS-EBt/MDR-AML; *p* < 0.01, Table 4). GA*TA2* mutation status was not significantly associated with DFS.

**Table 4:** Multivariate analysis of variables predicting Disease-free-survival (DFS) in a cohort of 65 patients with GATA2 deficiency and 404 patients without known predisposition syndrome.

	Relative risk	95 CI	*P*
Age at HSCT			
≥12 yrs. vs. <12 yrs.	1.1	[0.7–1.6]	n.s.
*GATA2* mutated			
yes vs. no	0.7	[0.4–1.3]	n.s.
Karyotype			
Monosomy 7 vs. normal	22	[1.2–3.9]	<0.01
Other vs. normal	1.6	[0.8–3.8]	n.s.
Other vs. monosomy 7	0.7	[0.4-1.3]	n.s.
Most advanced MDS type prior to HSCT			
MDS-EB vs. RCC	1.9	[1.0–3.4]	0.04
MDS-EBt/ MDR-AML vs. RCC	3.7	[2.2–6.3]	<0.01
MDS-EBt/MDR-AML vs. MDS-EB	2.0	[1.2–3.4]	0.01

*CI* confidence interval, *MDS* myelodysplastic syndrome, *HSCT* hematopoietic stem cell transplantation, *MDS-EB* MDS with excess blasts, *MDS-EBt* MDS with excess blasts in transformation, *RCC* refractory cytopenia of childhood, *MDR-AML* MDS-related acute myeloid leukemia, yrs years.

## Discussion

We present a comprehensive analysis of pediatric patients with GATA2 deficiency undergoing HSCT for MDS. Patients with inherited bone marrow failure disorders frequently demonstrate increased transplant-related toxicity and mortality upon undergoing HSCT, but whether this is true for pediatric patients with GATA2 deficiency has remained unclear. Several studies on HSCT in GATA2 deficiency reported small numbers of patients and/or patients of varying ages and heterogeneous disease characteristics [23]. For example, Parta reported the HSCT outcome of 22 patients with GATA2 deficiency conditioned with a busulfan-based regimen [10]. Although the results are encouraging, only four patients were under the age of 20 years, and infection was the indication in approximately half of the patients, rendering it difficult to interpret the results for pediatric GATA2-deficient patients with MDS.

In our study, patients with GATA2 deficiency transplanted for MDS had a similar outcome as compared to *GATA2*^wt^ patients. In multivariate analysis MDS type and karyotype but not GATA2 mutational status were significant variables for DFS, suggesting that the presence of the *GATA2* mutation is not a relevant risk factor.

We did not observe an unusually high rate of NRM or atypical non-infectious complications in *GATA2*^mut^ patients. A recent study reported a surprisingly high incidence of neurologic toxicities in 40% of transplanted *GATA2*^mut^ patients [24]. Here, we observed neurologic complications in four patients. Hofmann also noted an increased rate of thrombotic events. Although we did not observe a high incidence of thrombotic complications, several patients experienced transplant-associated thrombotic microangiopathy, and three of the four neurologic events were posterior reversible encephalopathy syndrome. This observation might indicate a defined endothelial vulnerability in *GATA2*^mut^ patients, consistent with the known role of GATA2 in the regulation of vascular integrity [25].

Interestingly, no mycobacterial infections were reported in this cohort. We did observe, however, a relatively high rate of fungal infections. HSCT performed in the past with limited surveillance and anti-fungal prophylaxis/treatments may have contributed to these findings. Overall, the frequency and distribution of different types of infections were consistent with general expectations in HSCT, with viral infections by far the most common complication.

Similar to organ toxicity, the rate of GvHD was not unusually high. In particular, cGvHD was observed in only 15 patients (24%).This is in contrast to the study by Parta [10] reporting cGvHD in 46% of patients, and points towards lower rates of GvHD in pediatric *GATA2*^mut^ patients.

EWOG-MDS HSCT recommendations stratify pediatric patients with MDS according to disease stage, karyotype and hematological presentation including bone marrow cellularity (Supplementary Figure 2). HSCT with a myeloablative regimen such as busulfan, cyclophosphamide, and melphalan is recommended for patients with increased blast count [26]. Patients with RCC and abnormal karyotype are also offered HSCT soon after diagnosis; we currently recommend a preparative regimen of thiotepa, treosulfan, and fludarabine. For patients with RCC and a normal karyotype, the decision to transplant depends on the hematological presentation. Transfusion dependent or neutropenic patients with RCC and hypocellular bone marrow are offered HSCT following a reduced toxicity regimen such as treosulfan and fludarabine, while in the absence of cytopenias patients with stable disease are generally offered a watch-and-wait strategy. The HSCT data presented here, in particular the highly similar outcome in *GATA*^mut^ as compared to *GATA*^wt^ patients with respect to OS, DFS, NRM and relapse, support the hypothesis that the currently recommended EWOG-MDS algorithm for therapy of pediatric MDS can also be applied to children with GATA2 deficiency. Although our series includes a limited number of patients with MDS and >20% blasts, the dismal outcome of this group with a high risk of relapse indicates the urgent need for evaluation of novel strategies including cytoreduction with modern agents such as CPX351 or venetoclax, and/or post-HSCT therapy with preemptive azacytidine and donor lymphocyte infusions.

The excellent outcome of HSCT in patients with *GATA2* germline disease, RCC morphology and normal karyotype raises the question whether these children should be offered HSCT once they have been diagnosed irrespective of their hematological presentation. The probability for progression to more advanced MDS is considerable, and early HSCT will spare patients cumbersome surveillance as well as the risk of inferior outcome of HSCT in more advanced disease. A similar issue arises for patients with GATA2 deficiency presenting with mild to moderate signs of immunedeficiency. Although the analysis presented here is limited to patients with MDS, the lack of evidence of increased transplant-related toxicity inherent to the *GATA2* germline mutation indicates that in young individuals with GATA2 deficiency the indication for HSCT can be based on the expected clinical course. Thus, preemptive HSCT might be an acceptable strategy. Our current approach is to perform a donor search as soon as GATA2 deficiency is diagnosed. In the absence of cytopenia, karyotypic abnormalities, increase in bone marrow blasts or clinically relevant immunedeficiency, we monitor the patient closely and consider a well-matched HSCT even without severe disease manifestations. Transplanting patients with GATA2 deficiency prior to the acquisition of severe infections or secondary organ damage, such as progressive pulmonary disease, is likely to increase long-term survival of adult patients with GATA2 deficiency.

One limitation of our study is that the presence of secondary mutations was unknown. It has previously been demonstrated that somatic *ASXL1* or RAS pathway mutations lead to leukemic transformation and inferior outcome [6, 27, 28]. In future prospective trials, secondary mutations need to be analyzed because they may serve as prognostic markers predicting the risk of relapse, and thus be crucial in guiding HSCT strategy.

In summary, our results indicate that pediatric patients with GATA2 deficiency are not at higher risk for HSCT-related complications or mortality compared to MDS patients without *GATA2* germline mutations. Overall, the relatively low rates of GvHD, infections, and organ toxicities suggest that standard HSCT protocols can be recommended. Considering the high mortality of untreated GATA2 deficiency and the high likelihood of developing MDS/AML, these data support a strategy of early preemptive HSCT in all pediatric patients with GATA2 deficiency.

## Supplementary information

Supplemental figure 1

Supplemental figure 2

Supplemental tables 1
