# Supplementary figures and images for: Hematopoietic stem cell transplantation in children and adolescents with GATA2-related myelodysplastic syndrome
Source: Bone Marrow Transplant. 2021 Jul 9;56(11):2732–41. doi: 10.1038/s41409-021-01374-y (PMC8563415; doi:10.1038/s41409-021-01374-y)

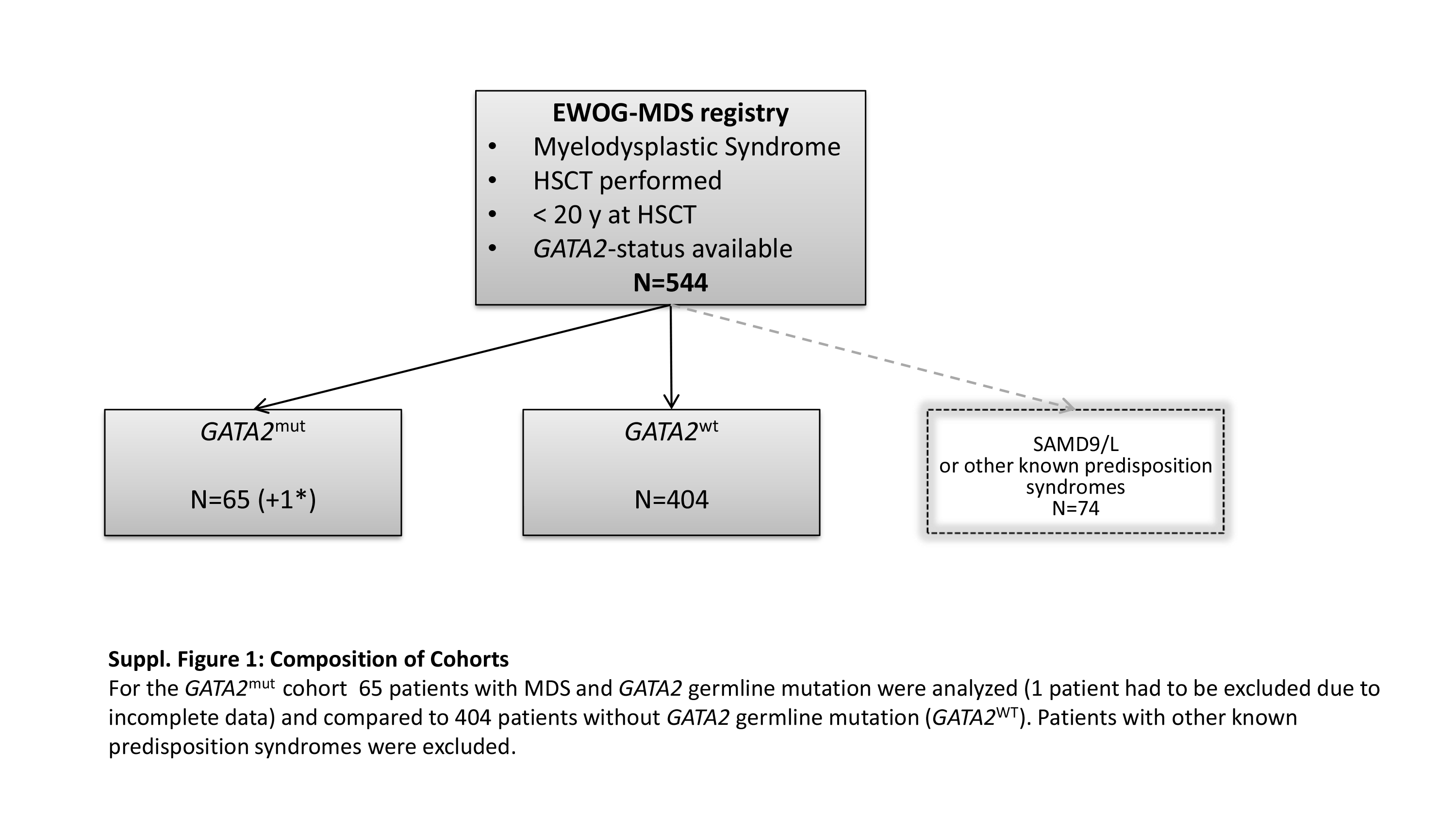

Supplement: Supplementary file 1 — Supplemental figure 1 [file 41409_2021_1374_MOESM1_ESM.tif]

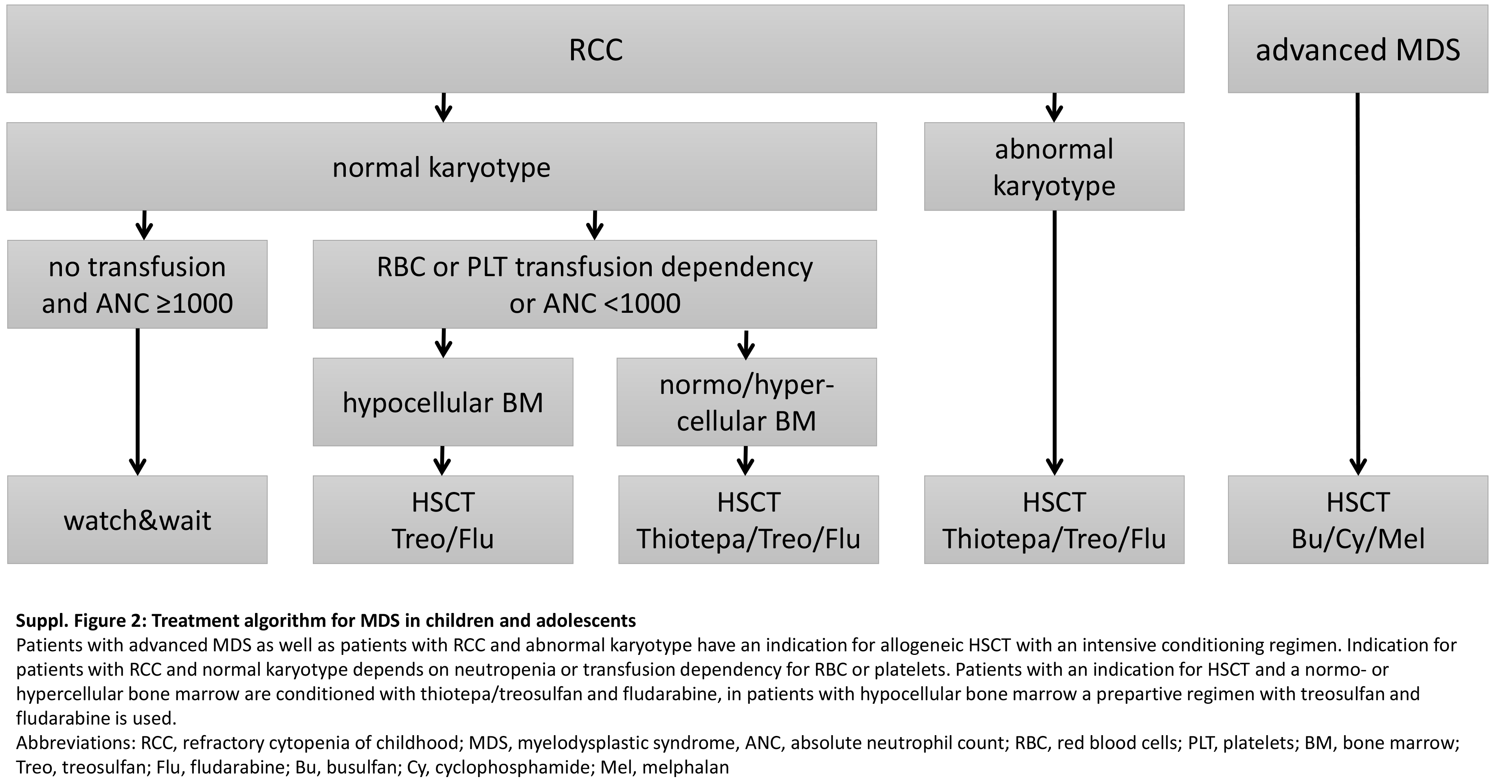

Supplement: Supplementary file 2 — Supplemental figure 2 [file 41409_2021_1374_MOESM2_ESM.tif]
