# Supplementary material for: Hematopoietic stem cell transplantation in children and adolescents with GATA2-related myelodysplastic syndrome
Source: Bone Marrow Transplant. 2021 Jul 9;56(11):2732–41. doi: 10.1038/s41409-021-01374-y (PMC8563415; doi:10.1038/s41409-021-01374-y)
Supplement: Supplementary file 3 — Supplemental tables 1 [file 41409_2021_1374_MOESM3_ESM.docx]

|  |  | **Total (65)**  **N (%)** | **MSD (17)**  **N (%)** | **MUD (30)** (HLA 10/10, 9/10)  **N (%)** | **UD (10)**  (HLA 6/6, 5/6, 8/10, incompl.)  **N (%)** | **MMFD (8)**  **N (%)** |
| --- | --- | --- | --- | --- | --- | --- |
| Engraftment |  | 65 (100) | 17 (100) | 30 (100) | 10 (100) | 8 (100) |
| Graft Failure sec |  | 0 | 0 | 2 | 0 | 2 |
| aGvHD | At Risk | 65 | 17 | 30 | 10 | 8 |
|  | °II-IV | 22 (34) | 5 (29) | 11 (37) | 6 (60) | 0 |
|  | °III/IV | 8 (12) | 2 (12) | 4 (13) | 2 (20) | 0 |
| cGvHD | At Risk | 62 | 16 | 30 | 9 | 7 |
|  | Limited/Extensive | 15 (24) | 4 (25) | 7 (23) | 2 (22) | 2 (29) |
|  | Extensive | 4 (6) | 1 (6) | 2 (6) | 1 (11) | 0 |

**Suppl Table 1 Engraftment and GVHD according to donor type in 65 patients with GATA2 deficiency transplanted for MDS**

Abbreviations: MSD, Matched sibling donor; (M)UD, (Matched) unrelated donor; MMFD, Mismatched family donor; sec, Secondary; aGvHD, Acute graft versus host disease; cGvHD, Chronic graft versus host disease

| **Item** | **Specification** |  | **DFS** | |  | **NRM** | |  | **Relapse** | |  |
| --- | --- | --- | --- | --- | --- | --- | --- | --- | --- | --- | --- |
|  |  | **N** | **Prob.** | **95%** | **p** | **Prob.** | **95%** | **p** | **Prob.** | **95%** | **p** |
| Number of patients |  | 65 | 70 | [58-82] |  | 14 | [8-26] |  | 16 | [8-29] |  |
| Year of HSCT | ≤2006 | 20 | 60 | [38-82] |  | 25 | [12-53] |  | 15 | [5-43] |  |
|  | 2007-2012 | 25 | 72 | [54-90] |  | 12 | [4-35] |  | 16 | [7-40] |  |
|  | ≥2013 | 20 | 83 | [65-100] | n.s. | 6 | [1-37] | n.s. | 12 | [3-43] | n.s. |
| Gender | Male | 34 | 69 | [52-82] |  | 15 | [7-33] |  | 16 | [7-37] |  |
|  | Female | 31 | 72 | [55-89] | n.s. | 13 | [5-33] | n.s. | 15 | [6-37] | n.s. |
| *GATA2* mutation | Truncating | 43 | 69 | [54-84] |  | 12 | [5-27] |  | 19 | [10-37] |  |
|  | Missense | 14 | 63 | [36-90] | n.s. | 21 | [8-58] | n.s. | 16 | [4-56] | n.s. |
|  | Non-Coding Intron | 4 |  |  |  |  |  |  |  |  |  |
|  | Synonymous | 3 |  |  |  |  |  |  |  |  |  |
|  | Other | 1 |  |  |  |  |  |  |  |  |  |
| Karyotype | Normal | 12 | 92 | [76-100] |  | 8 | [1-54] |  | 0 |  |  |
|  | Mono7 | 44 | 61 | [45-77] | 0.07 | 16 | [8-32] | n.s. | 23 | [13-41] | 0.08 |
|  | Other | 9 |  |  |  |  |  |  |  |  |  |
| Age at HSCT | <12 yrs | 19 | 67 | [55-89] |  | 11 | [3-39] |  | 23 | [10-54] |  |
|  | ≥ 12 yrs | 46 | 72 | [58-86] | n.s. | 15 | [8-30] | n.s. | 13 | [6-29] | n.s. |
| Donor | MSD | 17 | 82 | [64-100] |  | 12 | [3-43] |  | 6 | [1-39] |  |
|  | MUD | 30 | 74 | [55-93] |  | 7 | [2-26] |  | 19 | [9-43] |  |
|  | UD | 10 | 30 | [1-59] | 0.01 | 40 | [19-85] | 0.03 | 30 | [11-77] | n.s. |
|  | MMFD | 8 |  |  |  |  |  |  |  |  |  |
| MDS type at diagnosis | RCC | 36 | 76 | [61-91] |  | 14 | [6-31] |  | 10 | [3-30] |  |
|  | MDS-EB | 22 | 65 | [44-86] | n.s | 14 | [5-40] | n.s. | 21 | [9-50] | n.s. |
|  | MDS-EBt/MDR-AML | 7 |  |  | . |  |  |  |  |  |  |
| Most advanced MDS type prior to HSCT | RCC | 27 | 81 | [66-96] |  | 11 | [4-32] |  | 8 | [2-29] |  |
|  | MDS-EB | 23 | 73 | [54-92] |  | 13 | [5-38] |  | 14 | [5-41] |  |
|  | MDS-EBt/ MDR-AML | 15 | 49 | [21-77] | n.s. | 21 | [7-57] | n.s. | 31 | [14-70] | n.s. |
| BM blasts at HSCT | <5% | 34 | 79 | [65-93] |  | 9 | [3-26] |  | 13 | [5-31] |  |
|  | 5-19% | 19 | 73 | [52-94] | n.s. | 16 | [6-45] | n.s. | 11 | [3-42] | n.s. |
|  | ≥ 20% | 8 |  |  |  |  |  |  |  |  |  |
|  | Missing | 4 |  |  |  |  |  |  |  |  |  |
| Conditioning regimen | Busulfan-based | 35 | 69 | [53-85] |  | 15 | [6-33] |  | 16 | [7-36] |  |
|  | Treosulfan-based | 21 | 71 | [51-91] | n.s. | 10 | [3-36] | n.s. | 20 | [8-47] | n.s. |
|  | TBI-based | 5 |  |  |  |  |  |  |  |  |  |
|  | Other | 4 |  |  |  |  |  |  |  |  |  |
| Time from diagnosis to HSCT | <6 mo | 39 | 70 | [55-85] |  | 13 | [6-30] |  | 17 | [8-36] |  |
|  | ≥ 6 mo | 26 | 70 | [51-89] | n.s. | 15 | [6-38] | n.s. | 14 | [5-42] | n.s. |
| Stem cell Source | BM | 37 | 65 | [48-82] |  | 17 | [8-34] |  | 19 | [9-40] |  |
|  | PBSC | 19 | 78 | [59-97] | n.s. | 5 | [1-35] | n.s. | 16 | [6-46] | n.s. |
|  | PBSC (T-cell depleted) | 8 |  |  |  |  |  |  |  |  |  |
|  | CB | 1 |  |  |  |  |  |  |  |  |  |
| Gender match of donor | Match | 37 | 77 | [62-82] |  | 8 | [3-24] |  | 15 | [7-35] |  |
|  | No match | 27 | 60 | [40-80] | n.s. | 23 | [11-46] | n.s. | 17 | [7-41] | n.s. |
|  | Missing data | 1 |  |  |  |  |  |  |  |  |  |
| Non-hem. Features | No | 25 | 67 | [48-86] |  | 25 | [12-50] |  | 9 | [2-32] |  |
|  | Yes | 40 | 73 | [58-88] | n.s. | 8 | [3-22] | 0.08 | 20 | [10-39] | n.s. |
| Non-hem. Features | No | 25 | 67 | [48-86] |  | 25 | [12-50] |  | 9 | [2-32] |  |
|  | Yes, ID | 24 | 64 | [53-85] |  | 13 | [4-36] |  | 23 | [11-51] |  |
|  | Yes, Other | 16 | 84 | [63-100] | n.s. | 0 |  |  | 16 | [4-58] | n.s. |
|  |  |  |  |  |  |  |  |  |  |  |  |

**Suppl Table 2: Univariate analysis of 65 patients with GATA2 deficiency transplanted for MDS: DFS, NRM and Relapse according to patient and disease characteristics, and transplantation procedure**

Abbreviations: DFS, Disease free survival; NRM, Non-relapse mortality; MSD, Matched sibling donor; MUD, Matched unrelated donor; UD, Unrelated donor; Haplo, Haploidentical; MDS, Myelodysplastic syndrome; RCC, Refractory cytopenia of childhood; MDS-EB, MDS with excess blasts; MDS-EBt, MDS with excess blasts in transformation; MDR-AML, MDS-related acute myeloid leukemia; TBI, Total body irradiation; BM, Bone marrow; PBSC, Peripheral blood stem cells; CB, Cord blood; ID, immunedeficiency; yrs, years; mo, months

|  |  | **GATA2deficiency N (%)** | **No known predisposition syndrome**  **N (%)** | **p** |
| --- | --- | --- | --- | --- |
| Number of patients |  | 65 | 404 |  |
| Gender, male/female |  | 34/31 (52/48) | 238/166 (59/41) | n.s. |
| Age at HSCT, years | (median, range) | 13.5 (4.6-19.0) | 10.6 (1.1-19.9) | <0.01 |
| Most advanced MDS type  prior to HSCT | RCC | 27 (42) | 290 (72) | <0.01 |
|  | MDS-EB | 23 (35) | 57 (14) |  |
|  | MDS-EBt/ AML | 10/5 (23) | 28/29 (14) |  |
| Karyotype | Monosomy 7 | 44 (68) | 47 (14) | <0.01 |
|  | Der (1;7) | 4 (6) | 1 (0) |  |
|  | Trisomy 8 | 4 (6) | 24 (7) |  |
|  | Normal | 12 (19) | 249 (73) |  |
|  | Other | 1 (2) | 20 (6) |  |
| Most advanced MDS type prior to HSCT according to Karyotype |  |  |  | <0.01 |
| RCC | Monosomy 7 | 12 (44) | 19 (8) |  |
|  | Normal | 10 (37) | 199 (86) |  |
|  | Other | 5 (19) | 13 (6) |  |
| MDS-EB/ MDS-EBt/ MDR-AML | Monosomy 7 | 32 (84) | 28 (26) |  |
|  | Normal | 2 (5) | 50 (46) |  |
|  | Other | 4 (11) | 32 (29) |  |

**Suppl Table 3: Comparison of patient and disease characteristics of 65 patients with GATA2 deficiency and 404 patients without known underlying MDS predisposition syndrome transplanted during the same time period**

Abbreviations: MSD, Matched sibling donor; UD, Unrelated donor; MMFD, Mismatched family donor; sec, Secondary; aGvHD, Acute graft versus host disease; cGvHD, Chronic graft versus host disease
